# Deep-learning framework for fully-automated recognition of TiO_2_ polymorphs based on Raman spectroscopy

**DOI:** 10.1038/s41598-022-26343-3

**Published:** 2022-12-19

**Authors:** Abhiroop Bhattacharya, Jaime A. Benavides, Luis Felipe Gerlein, Sylvain G. Cloutier

**Affiliations:** Department of Electrical Engineering, École de technologie supérieure, 1100 Notre-Dame West, Montreal, QC H3C 1K3 Canada

**Keywords:** Characterization and analytical techniques, Electrical and electronic engineering, Computer science

## Abstract

Emerging machine learning techniques can be applied to Raman spectroscopy measurements for the identification of minerals. In this project, we describe a deep learning-based solution for automatic identification of complex polymorph structures from their Raman signatures. We propose a new framework using Convolutional Neural Networks and Long Short-Term Memory networks for compound identification. We train and evaluate our model using the publicly-available RRUFF spectral database. For model validation purposes, we synthesized and identified different TiO_2_ polymorphs to evaluate the performance and accuracy of the proposed framework. TiO_2_ is a ubiquitous material playing a crucial role in many industrial applications. Its unique properties are currently used advantageously in several research and industrial fields including energy storage, surface modifications, optical elements, electrical insulation to microelectronic devices such as logic gates and memristors. The results show that our model correctly identifies pure Anatase and Rutile with a high degree of confidence. Moreover, it can also identify defect-rich Anatase and modified Rutile based on their modified Raman Spectra. The model can also correctly identify the key component, Anatase, from the P25 Degussa TiO_2_. Based on the initial results, we firmly believe that implementing this model for automatically detecting complex polymorph structures will significantly increase the throughput, while dramatically reducing costs.

## Introduction

Vibrational spectroscopy involves specific optical techniques including Fourier-transform infrared (FTIR), Brillouin scattering and Raman spectroscopy^1^. Classification models for vibrational spectroscopy data can map input objects (spectra) to desired outputs (class assignments)^2^. While building such a classification tool is a challenging task, it would benefit a wide range of application sectors including semiconductors, pharmaceutical, polymers, forensics, environmental and food sciences, as well as medicine^3,4^. Also, vibrational spectroscopy data often requires additional and complex pre-processing steps before chemometric measurements^5^. Several data pre-processing approaches are used to improve the robustness and accuracy of subsequent multivariate analysis and to increase the interpretability of the data by providing adjustments for the challenges associated with spectral data acquisition^5^. However, pre-processing methods are often proprietary, or they can vary significantly depending on the objective of the study and the technique. These expert guided pre-processing pipelines are brittle and are based on empirical evidence^6^. As a result, a pre-processing technique suitable for a given model or material system might decrease the performance of another^7^. The effects of a given pre-processing technique also varies significantly with the data sample, often requiring a trained expert’s intervention. Thus, we believe it is important to remove the non-standard expert guided pre-processing of signals to ensure better generalization.

Recent advances in the development of instruments coupled with more powerful compute resources and tools have brought Raman spectroscopy from a research laboratory-based method to a widely-used method in a diverse set of fields. Portable or handheld systems are currently helping clinicians in the detection of cancerous cells and in the identification of unknown chemical substances^8–11^ Raman spectroscopy provides unique fingerprints pertaining to individual molecules and compounds and it requires minimal sample preparation. Similarly, Raman spectroscopy can detect and characterize defect states that significantly affect the material’s properties^12,13^. For instance, the presence of surface defects in metal-oxides allow anions and cations to assume a variety of charged states contributing to photocatalysis^14^, corrosion protection^15^, sensors^16^, microelectronics^17^, magnetic recording devices^12,15^ and micro-porous materials^18^.

Spectroscopy techniques can be subject to individual interpretations. During manual pre-processing, human bias might be added to the data, which can impact the results or their interpretations^7^. Thus, there is an urgent requirement to develop more robust computational tools, which would help users rapidly analyse the spectrum patterns in a more uniform and reliable (unbiased) manner. Traditional methods require a highly-skilled expert to correctly identify the compound and/or structural properties from the Raman spectroscopy measurements. Convolutional Neural Networks (CNNs) have been widely used to extract feature patterns in complex high-dimensional data. Thus, they can reduce the need for brittle manual pre-processing by highly-skilled experts^19^. Automated real-time CNN models enable professionals from a diverse set of fields to use Raman spectroscopy to identify increasingly complex compounds from more affordable (lower-resolution) instruments with minimal preprocessing of data.

In this work, we propose a powerful deep-learning framework to accurately identify compounds from their Raman spectra. Using the proposed model, we achieve a Top-1 accuracy of 99.12% and a Top-5 accuracy of 99.30% on the public RRUFF Dataset. To support our findings, we present experimental validations using our model to accurately identify both Rutile and Anatase in the synthesized samples. Most importantly, we establish that the proposed model does not require any additional expert guided proprietary data pre-processing to achieve accurate identification. This leads to better cost efficiencies and standardisation.

## Related works

Several researchers worked on the development of spectral-matching algorithms^20,21^. These algorithms identify the similarities between a reference spectrum and the samples in an iterative manner^20^. These techniques can be classified into *unsupervised* or *supervised* methods^22^.

The unsupervised methods tend to reduce the number of dimensions using Principal Component Analysis (PCA), and then use a similarity or distance-based algorithm like K-Nearest Neighbour (KNN) to identify homogeneous clusters in the spectral data^23^. The distance-based algorithms maximize the similarity within a class and reduce the inter-group similarity^24^. The reference sample and technique used for dimensionality reduction have a significant impact on the performance of the unsupervised method^25^. In contrast, the similarity-based methods either compare the maximum peaks or the full spectrum^26^. Then, a wide selection of distance metrics such as Euclidean distance and least squares can be used to calculate similarity^27^. These algorithms require feature engineering and a reference database to identify compounds. Several researchers report that the feature engineering approach is not robust^28,29^. Feature engineering is usually specific to a dataset and it cannot be easily applied across different datasets. Furthermore, it provides a gateway for human bias to be introduced in the model and it still requires a highly-skilled practitioner to analyse the results.

In contrast, supervised methods minimize the error between the predicted label and the ground truth label during training^30^. As such, they require a labeled corpus of data for the training. The algorithm extracts the features from the input spectrum. It then makes a prediction of the compound class based on these features. Subsequently, it quantifies the difference between the ground truth and the predicted value using a measure for similarity. This loss can then be optimized using different optimisation algorithms. Several researchers have applied traditional machine-learning (ML) techniques to classify Raman spectra^31,32^. Support vector machines (SVMs) were used with limited success^33,34^. Unfortunately, the performance of the SVMs deteriorates rapidly as the number of classes increases^35^.

Fully-connected dense network were recently applied to Raman spectra analysis^36^. However, dense networks are unable to extract features from the spectrum and have a large number of parameters leading to data overfit^37^. Researchers also explored the use of 1D CNNs to analyze Raman spectra^35,38,39^. Their results suggest that convolutional networks can accurately classify the spectra with minimal pre-processing treatment. An accuracy of 93.3% was reported on the pre-processed samples of the RRUFF dataset^35,40^. Building on these promising results, a two-step model was trained for identifying each compound from their spectra and then performing the identification of compounds from a mixture with a classification accuracy of 98.8%^41^. However, this approach is not scalable to a mixture of many compounds.

More recent transfer learning and data augmentation techniques are now widely used to reduce overfitting and improve the performance of the models^42^. Augmented spectral datasets were generated by adding various offsets, slopes and multiplications on the vertical axis^43^. Meanwhile, transfer learning was also performed by training the network on a spectral database and using the model to predict the observations from a different database^44^. This approach yields an accuracy of 88.4% for the unprocessed data and 94% for the processed data.

Raman spectrum analysis usually requires baseline correction prior to spectral matching. There is a wide range of methods for baseline correction, such as a least-squares polynomial curve fitting for the subtraction of the baseline^45^. The literature provides a comprehensive survey of baseline and correction methods^46^, and a comprehensive overview of the applications of convolutional neural networks to vibrational spectroscopy measurements^46^.

In this work, we implement a deep-learning convolutional model to identify compounds from their Raman spectra. Using this approach, we perform an experimental verification of the model using anatase and rutile TiO_2_ polymorphs.

The proposed model is an end-to-end framework, which does not require any additional proprietary pre-processing. To the best of our knowledge, this is the first model using a combination of Long Short Term Memory networks (LSTM)^47^ and convolutional neural networks (CNNs) for Raman spectra analysis. Most importantly, the ablation study indicates that LSTM incorporation leads to a significant improvement in classification accuracy. In time, we firmly believe such real-time machine learning-assisted compound identification and analysis will help rapidly and more accurately identify the mineral and chemical compounds.

## Materials and methods

**Figure 1:**
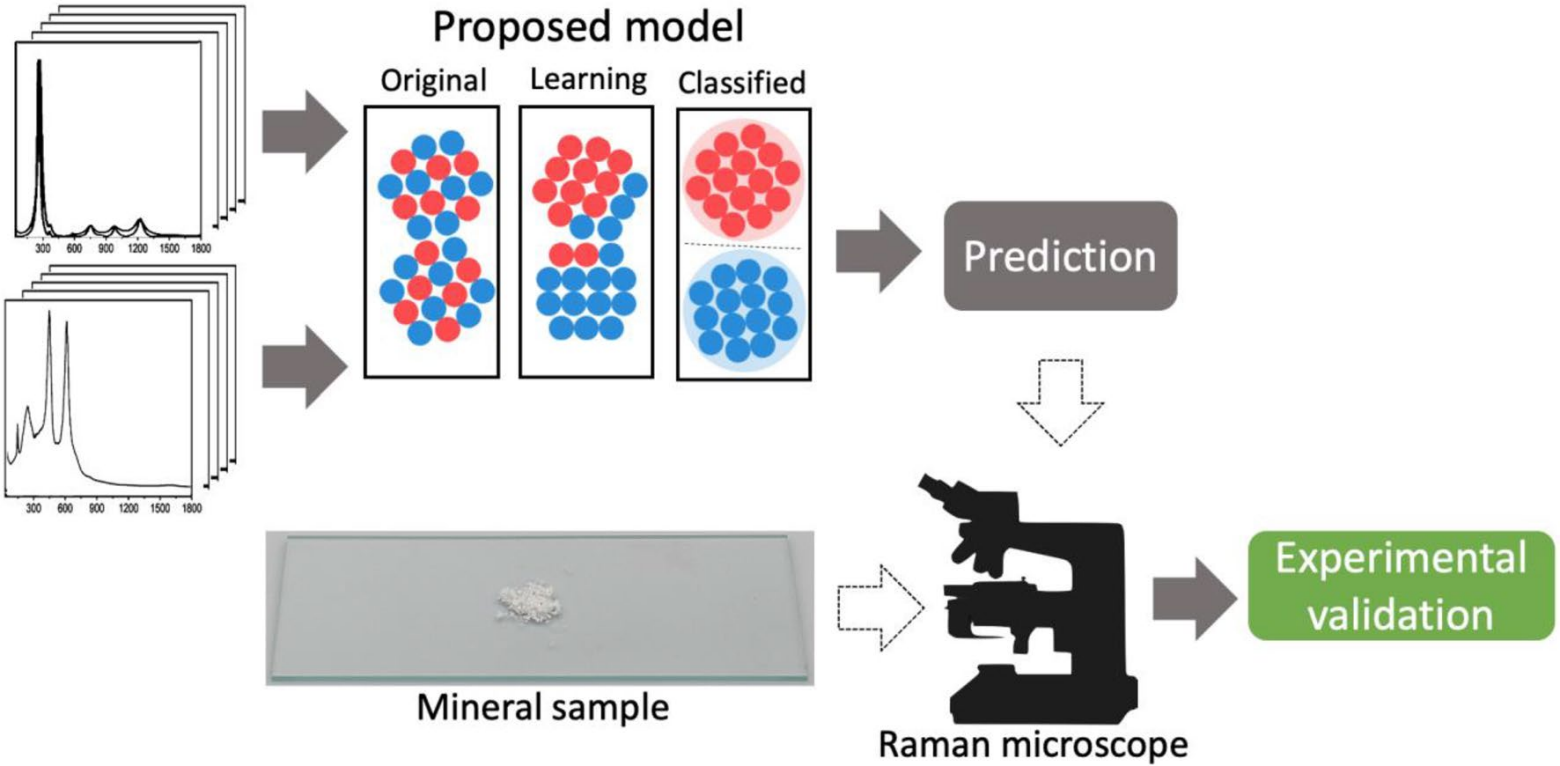
Schematic overview of the process flow.

This section gives a brief overview of the various models used in the paper. The Fig. 1 presents a high-level overview of the methodology. The proposed model is trained using the Raman spectra from the database. The trained model takes a Raman spectrum as an input and identifies the mineral. We compare the model prediction against real world data to validate the model outcome. The section presents a detailed overview of the training and experimental methodology.

### Proposed model architecture

The input to the CNN in application to Raman spectrum classification is one dimensional and it contains the entire spectrum. Hence, we trained one dimensional convolutional kernel in our CNN. We use a ReLU activation for the convolutional layers:

1 $$f\left(x\right)\left\{\begin{array}{ll}x & \quad x\ge 0\\ 0 & \quad otherwise\end{array}\right.$$

The “Ablation studies” section will compare results using other activation functions. A convolutional layer can be expressed as follows:

2 $${y}^{j}=F\left({b}^{j}+\sigma {k}^{ij}*{x}^{j}\right)$$

where x^i^ and y^j^ are the ith input and jth output map, respectively. K^ij^ is a convolutional kernel between the maps i and j, * denotes the convolution operator and b^j^ is the bias parameter of the jth map. The convolutional layer is followed by a max-pooling layer, in which each neuron in the output map y^i^ pools over an SXS non-overlapping region in the input map x^i^.

Formally, the max-pooling operation is described as:

3 $${y}_{j}^{i}= {\underset{0<m<s}{\mathrm{max}}}x_{j \cdot s+m}^{i}$$

The output of the max-pooling layer is fed to a Long Short-Term Memory (LSTM) layer to process the one-dimensional sequence. The LSTM network has a cell state, which can be used to remember the previous timestamp. The output of the LSTM is flattened and processed using fully-connected dense layers.

We use ReLU as the non-linear activation for the fully connected layers. The model has four 1D Convolutional layers with a kernel size of 2 and *same* padding. The max pooling layers have a pool size of 2 and a stride of 2. The SoftMax operates as a squashing function that re-normalises the K dimensional input vector *z* of real values to real values in the range [0,1] that sum to 1 specifically,

4 $$\sigma {\left(z\right)}_{j }= \frac{{e}_{j}^{z}}{\sigma {e}_{k}^{z}}$$

**Figure 2:**
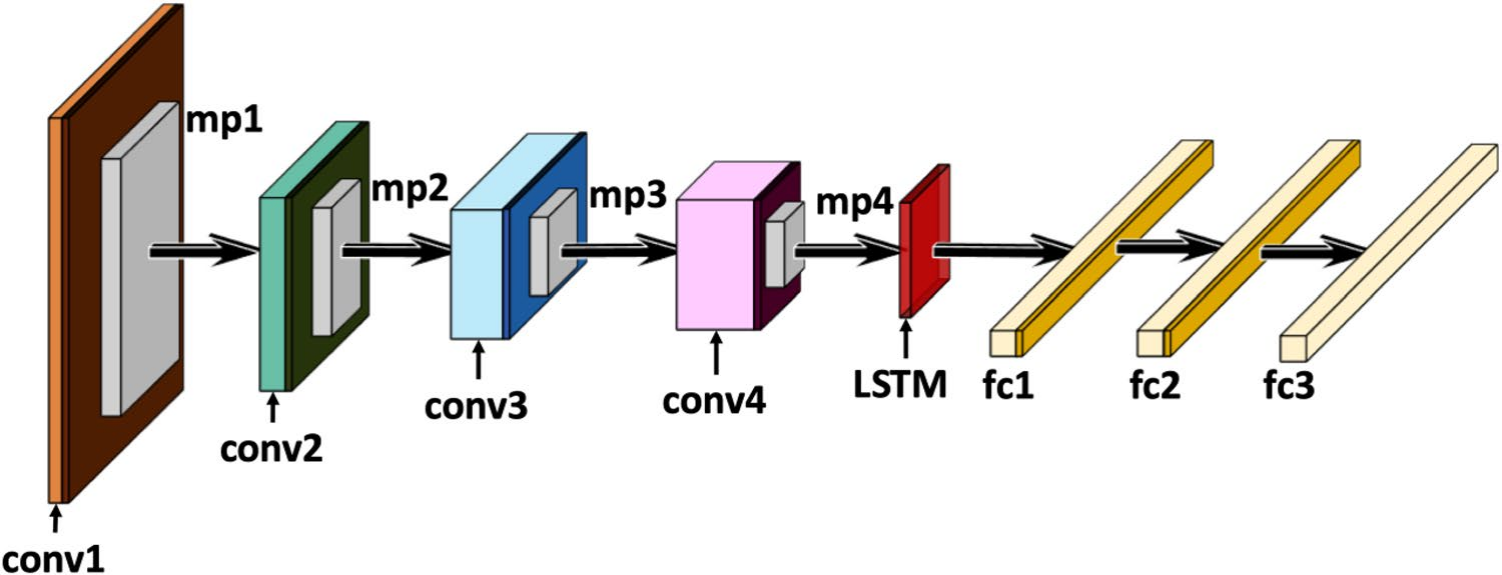
Proposed Model Architecture. Dark coloured boxes indicate a ReLU activation function at the end of a convolution "conv" layer. The max-pooling operation is represented by the nomenclature "mp" and the fully connected dense layers are denoted by “fc”.

To avoid overfitting, we apply a dropout after the first and second fully-connected layer^48^. The block diagram in Fig. 2 shows the structure of the proposed network. A detailed description of the model and the hyperparameters is provided in the supplementary information in the section “Model Diagram”.

### Model training

The training of the model is performed using RMSProp algorithm^49^, which is a variant of the stochastic gradient descent using 100 epochs with learning rate of 1e -3 and β = 0.9. The layers are first initialised from a Gaussian distribution with a zero mean and variance equal to 0.05. The framework is implemented using the Tensorflow library version^50^. We use a Tesla T4 graphics processing unit (GPU) to run all experiments.

### RRUFF dataset

The project uses the public RRUFF dataset^40^. The RRUFF project provides access to X-ray diffraction patterns, Raman spectroscopy, Fourier-transform infrared (FTIR) spectroscopy, references, photographs and characterization data of different minerals. The database is constantly reviewed and updated to ensure the quality and durability of data. The database contains the spectral information of 3527 (over 70%) of the 4985 known minerals. Public access is provided with reviewed and validated characterizations of 2128 of those minerals.

The dataset contains the Raw and baseline-corrected Raman spectra of mineral polymorphs. The Raman spectra of the minerals in the project are acquired using several spectrometers. Some of the spectrometers are: Downs spectrometer with 514 nm laser, Thermo Almega XR with 532 and 785 nm lasers, Kaiser Optical Systems HoloProbe 785 and a Renshaw microRaman system for work at 514.5 and 780 nm. Based on the quality of sample collection, the samples are divided into excellent, fair, poor and unrated. These are further divided by the orientation and the processing. A detailed description of the different splits has been provided in the supplementary information in the section “*Analysis of RRUFF Dataset”*.

### Evaluation metrics

Classification models use true positives and false positives for *precision* and *recall* assessment:

5 $$Precision =\frac{True \; Positives}{True \; Positives+False \; Positives }$$

6 $$Recall = \frac{True \; Positives }{True \; Positives+False \; Negatives }$$

The *Precision* value indicates the model's positive predictive value or the ability to avoid false positives (here, this would mean predicting it is a given mineral when it is not). Meanwhile, the Recall value indicates the model's true positive rate or *sensitivity* or the ability to avoid false negatives (here, this would mean predicting it is not a given mineral when it is). Balancing the *Precision* and *Recall* in the context of a specific application is one of the main challenges facing any machine-learning developer^51^.

### Methodology

We have used a training methodology to train the model and experimental evaluation to predict the TiO_2_ polymorphs from synthetized samples.

#### Training methodology

We first randomly divide the whole dataset into training (80%), validation (10%) and test (10%) sets. The stochastic gradient descent (SGD) optimizer is used for most of the experiments. Additional experiments were performed using the Adam and AdamW optimizers^49^. However, we find that a variant of SGD consistently produces the best results. The same behavior was previously reported in the literature^52^. The hyperparameters are fine-tuned for each model and the detailed list of hyperparameters associated with each model is provided in the supplementary section. We trained our model using various Convolutional network architectures and activation functions for comparison.

#### Experimental evaluation

TiO_2_ polymorphs play an essential role in diverse applications ranging from photo-catalysis to energy-harvesting^53,54^. Furthermore, researchers have shown that it is possible to have an enhanced room temperature photo conversion by utilising a defect-rich synthesis of TiO_2_^55^. Rapid characterization of TiO_2_ polymorphs is crucial for several applications such as additive manufacturing^56^. To test our model using real data from our laboratory, we synthesized standard white anatase, defect rich anatase and rutile TiO_2_, by sol gel chemistry using a protocol described in the literature^57^. In this context, we can evaluate the performance of our model at predicting and identifying crystalline TiO_2_ phases in a real empirical scenario. We focus on the Top-5 model predictions for each experimental evaluation in the bar charts presented below. The high Top-5 accuracy of our model indicates that for most instances the correct mineral lies within the top 5 predictions of the model. The model outputs for all experiments are presented in detail in the Supplementary Information.

### Processed dataset

In the real world, a Raman spectrum might be corrupted by a poor focus (when performed through a microscope), a fluorescence background (from the sample), CCD background noise (from the detector), Gaussian noise, stray-light cosmic noise (from the spectrometer)^7^. All these phenomena can distort the Raman spectrum. Whenever possible, it is essential to remove these undesirable artefacts in order to extract the best information. In addition to these corrections, it is sometimes necessary to correct for varying sampling geometries and highly redundant variables^7^. The RRUFF database provides both raw and processed data.

First, we train our model using the processed data for benchmark comparisons. This is consistent with the state-of-the-art, where researchers usually compare their model performances using the processed RRUFF data to evaluate their models. The processed RRUFF dataset has 5681 samples.

### Raw dataset

Pre-processing the Raman spectrum is an essential part of the analysis process. The use of specialised software to pre-process the samples significantly increases the cost. Moreover, samples can be pre-processed separately by different experts leading to significant costs, delays and discrepancies from one expert to another. Also, this pre-processing is not versatile, prone to human errors and cannot be easily adapted to different environments. During pre-processing, information can be incorporated into or removed from the data, preventing generalization and hampering its performance. On the other hand, deep neural networks are data hungry and training the network on a larger dataset is often necessary to allow better generalization of the model^58^.

**Figure 3:**
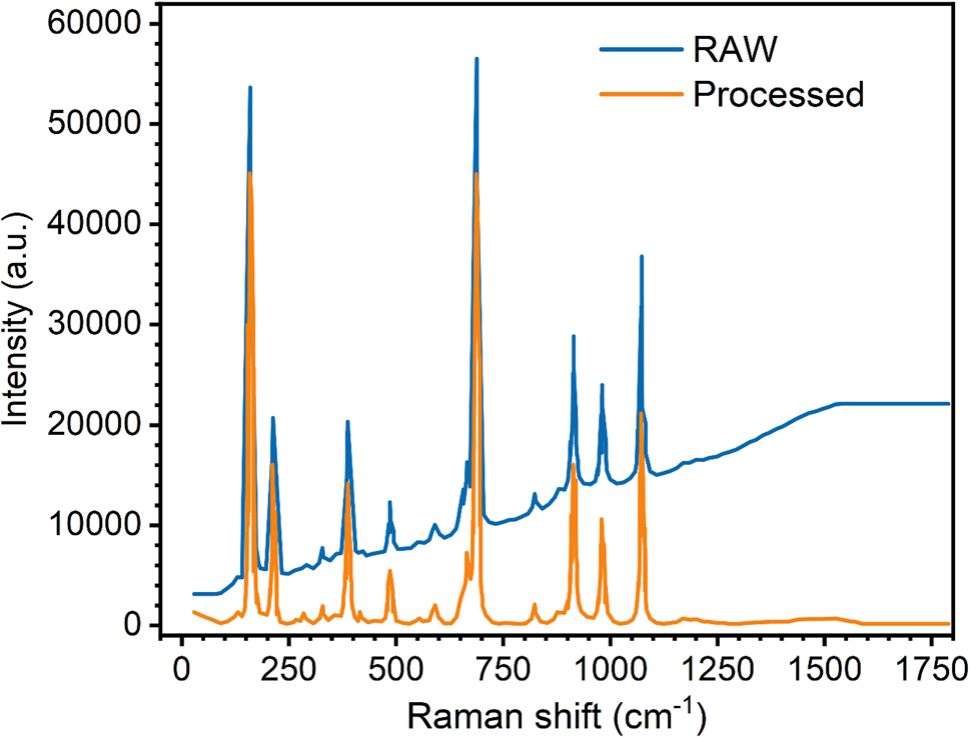
The effect of processing the raw data using Savitzky Golay filter and background subtraction using penalized least squares. The processed data does not suffer from the effects of the noise and fluorescence.

We added a processing step in our framework to treat the raw data before passing it into the neural network. We first use the Savitzky Golay filter^59^ to remove the noise from the sample and subsequently, use penalized least square method for subtracting the background noise^60^. This enables the use of raw oriented samples available in the RRUFF dataset for additional training. Thus, we can directly identify the compounds from the raw spectra without using any additional expert guided proprietary data pre-processing. Figure 3 shows a typical example of pre-processing the raw data using Savitzky Golay filter and background subtraction using penalized least squares. The processed data does not suffer from the effects of the noise and fluorescence.

### Synthesis of experimental samples

For the experimental evaluation, first we synthesize the mineral samples as per the methodology described below. Subsequently, we obtain our own Raman spectroscopy results from the synthesized samples using the WITec Alpha 300 confocal Raman microscope using a 532 nm laser excitation through a 10× microscope objective. The software provides a series of correction features. We first remove the cosmic ray noise and then the harmonic peaks. Subsequently, we perform background (baseline) subtraction. We repeat the same processing for each of the individual samples.

#### Synthesis of standard white anatase

To prepare the standard white anatase, we mix 28.8532 g of ethanol (Sigma-Aldrich 493511) with 10.8604 g of titanium (IV) butoxide (Sigma-Aldrich 244112). This solution is stirred for 40 min. Then, the hydrolysis reaction is triggered by adding 0.84 mL of deionized water. The precipitation of the amorphous white TiO_2_ occurs within the first few seconds after the reaction is triggered. This mixture is aged for 72 h and then the solvent is evaporated at ambient temperature. The amorphous TiO_2_ powder is annealed at 550 °C for two hours to yield standard white anatase. In the same way, rutile crystalline phase is obtained by annealing the amorphous TiO_2_ at 1100 °C for 2 h.

#### Synthesis of defect-rich black anatase

To prepare the defect-rich black anatase, 28.9024 g of ethanol (Sigma-Aldrich 493511) are mixed with 1.4748 g of acetylacetone (Sigma-Aldrich 10916). This solution is stirred for 20 min. Then, 10.8512 g of titanium (IV) butoxide (Sigma-Aldrich 244112) are added and stirred for 40 min more. The hydrolysis reaction is triggered by adding dropwise 0.84 mL of deionized water. This solution is covered with parafilm to restrict the income of air and avoid contamination. Finally, the resulting mixture is stirred for 120 min and then aged for 8 months. During the aging time, the ethanol evaporates to precipitate a defect-rich TiO_2_ amorphous powder.

These defect-rich TiO_2_ amorphous powders are annealed at 450 °C for 2 h to obtain defect-rich black anatase. In the same way, modified rutile crystalline phase is obtained by annealing at 1100 °C for 2 h.

#### Commercial Degussa TiO_2_ powder

Degussa TiO_2_ (Sigma-Aldrich 634662) was purchased and used as received. The crystalline composition of Degussa is typically around 78% anatase, 14% rutile and around 8% of amorphous TiO_2_^61^.

## Results and discussions

The results in the Table 1 show that our method can accurately identify pure compounds from the Raman spectra. The cited metrics reported in the Table are based on the processed spectra in the “excellent oriented” and “excellent unoriented” subsets of the RRUFF database. Furthermore, the cited authors have split the dataset based on the number of samples in the same category. When deploying the model in a real-world scenario, it is likely to expect that the dataset will contain a mix of different data qualities. Thus, we have reported the results on a mix of excellent, fair, poor and unrated oriented processed spectra without any segmentation. Most importantly, our model is able to achieve a similar accuracy on the un-processed data. Compared with the state-of-the-art, our proposed model achieves an increase in accuracy of 2% in Top-1 accuracy. Indeed, the model achieves a Top-1 accuracy of 99.12% and a Top-5 accuracy of 99.30%.

**Table 1:** The table benchmarks our model against the state-of-the-art models from the literature.

Model	Top-1 accuracy (%)	Top-5 accuracy (%)	Precision (%)	Recall (%)
RamanNet (Ibtehaz et al.)^62^	97.92	99.50	–	–
DeepCNN (Liu et al.)^35^	96.01	99.44	96.60	96.01
DeepCID (Fan et al.)^41^	96.79	99.55	97.18	96.79
TLCNN (Zhang et al.)^44^	95.83	99.62	96.53	95.83
CNN (Sang et al.)^63^	97.72	99.84	97.99	97.72
DRCNN (Zhou et al.)^64^	97.98	99.81	98.13	97.98
CNN1D	96.83	98.94	97.18	96.83
CNN-LSTM (proposed) For pre-processed data	99.12	99.30	99.30	99.12
CNN-LSTM (proposed) For unprocessed (raw) data	98.61	99.31	98.78	98.61

The bold values indicate the results from our proposed model for the processed and raw spectra.

The Top-5 accuracy metric indicates that the correct mineral is 99.30% of the time in the five (5) most likely candidates identified by the model. The Top-1 accuracy metric indicates that the correct mineral is 99.12% the most probable candidate identified by the model. In this case, the Top-1 accuracy is obviously equal to the recall value, while the precision value is slightly higher at 99.30%. As should be expected, the raw unprocessed data yield a similar Top-5 accuracy of 99.31% but a slightly lower Top-1 accuracy of 98.61%.

The results clearly show that our model is able to identify the compounds from their Raman spectra. The training dataset includes multiple spectra to characterize the same mineral. These spectra have been acquired by different operators at different institutes, using different instruments, environments and sample preparation conditions. This makes the model resilient to minor changes in the pattern and makes the model generalize to the test data.

### Misclassification

There are a few cases where the model is not able to accurately identify a compound. In this section, we will investigate these rare instances in further details to better understand the mechanisms involved. These misclassifications almost exclusively occur when the model associates a low probability with all the predictions. This suggests that the model is confused as it shows a low level of confidence in its prediction. To mitigate these cases, we suggest that the users conduct an expert evaluation when the model yields a low probability.

We also believe that increasing the training data by including a wider variety of minerals will mitigate this type of misclassification. This may be also caused by a spectrum which was not acquired using optimal parameters. Often in such cases, the correct prediction is amongst the list of prediction albeit with a low probability. This is consistent with measuring a Top-5 accuracy (99.30%) slightly higher than the Top-1 accuracy (99.12%).

However, there are some very rare instances where our model associated a high probability (suggesting a high level of confidence) to an incorrect prediction. This usually indicates a generalization problem, where the distribution of the test samples is very similar to the distribution of another sample in the training data^65^. In layman’s terms, this occurs when the distribution of a given test spectra is very similar to a different mineral from a training spectrum. For example, this can occur if we have a single low-quality Raman spectrum (very noisy or where the crystal structure is not clearly defined) present in the dataset. Impurities in the sample may also lead to shifts in the peak or changes in distribution, thus affecting the model. We have provided a specific example of such a misclassification error in the supplementary information, which is consistent with this interpretation.

### Ablation studies

The following section presents ablation studies performed on the different constituents of the model. An ablation consists in removing and/or modifying certain components of the model and observing the effect on the model performances.

#### LSTM module

LSTMs are widely used in the literature to analyse time-series data^66^. It helps the model extract features from sequential datasets. In our model, LSTM works in the latent space across feature maps. We observe a significant drop of 2.29% in Top-1 accuracy if we remove the LSTM module altogether (CNN1D). Therefore, the LSTM module is crucial for improving the model performance. However, we observe that using a Bidirectional LSTM (CNN-BiLSTM) leads to a major deterioration in performances. This can be intuitively expected because the ordering and distribution of the peaks defines the Raman spectrum. We also stacked multiple LSTM layers (CNN-2LSTM), which also leads to a significant reduction in performances. The addition of layers adds levels of abstraction of input observations over time, which may lead to grouping similar observations over time^67^. This can confuse the model between similar spectra and is detrimental to the performance of the model. The Table 2 compares the results using different LSTM architectures or no LSTM at all (CNN1D).

**Table 2:** The table compares the performance using different or no LSTM architectures.

Model	Top1 accuracy (%)	Top5 accuracy (%)	Precision (%)	Recall (%)
CNN1D (no LSTM)	96.83	98.94	97.18	96.83
CNN-BiLSTM	33.39	65.73	74.32	9.67
CNN-2LSTM	12.56	39.02	83.33	1.76
CNN-LSTM	99.12	99.30	99.30	99.12

The bold values indicate the results from our proposed model for the processed and raw spectra.

#### Activation function

We also compare the performances of the model using different activation functions. We change the activation function for the convolutional layer and dense layers. We observe that using a ReLU activation function gives the highest accuracy. As per our observations, the Tanh, LeakyRelu, Selu, Swish and GeLU activation functions all show lower performances compared to the ReLU activation. Very interestingly, we get a significant performance degradation using the sigmoid activation function. Since the derivative of the sigmoid function is always less than one, we believe multiplying the gradient across layers may diminish the signal and create a vanishing gradient problem^68^. The Table 3 compares the model performance using different activation functions.

**Table 3:** The table compares the performance of different activation functions.

Activation function	Top1 accuracy (%)	Top5 accuracy (%)	Precision (%)	Recall (%)
ReLU	99.12	99.30	99.30	99.12
Tanh	98.6	99.3	99.5	98.4
LeakyReLU	98.8	99.1	99.1	99.3
Swish	99.0	99.3	99.8	99.0
Gelu	97.2	99.1	99.5	99.1
Selu	98.8	99.3	99.3	98.8
Sigmoid	74.2	95.3	92.0	46.6

The bold values indicate the results from our proposed model for the processed and raw spectra.

### Experimental outcomes

**Figure 4:**
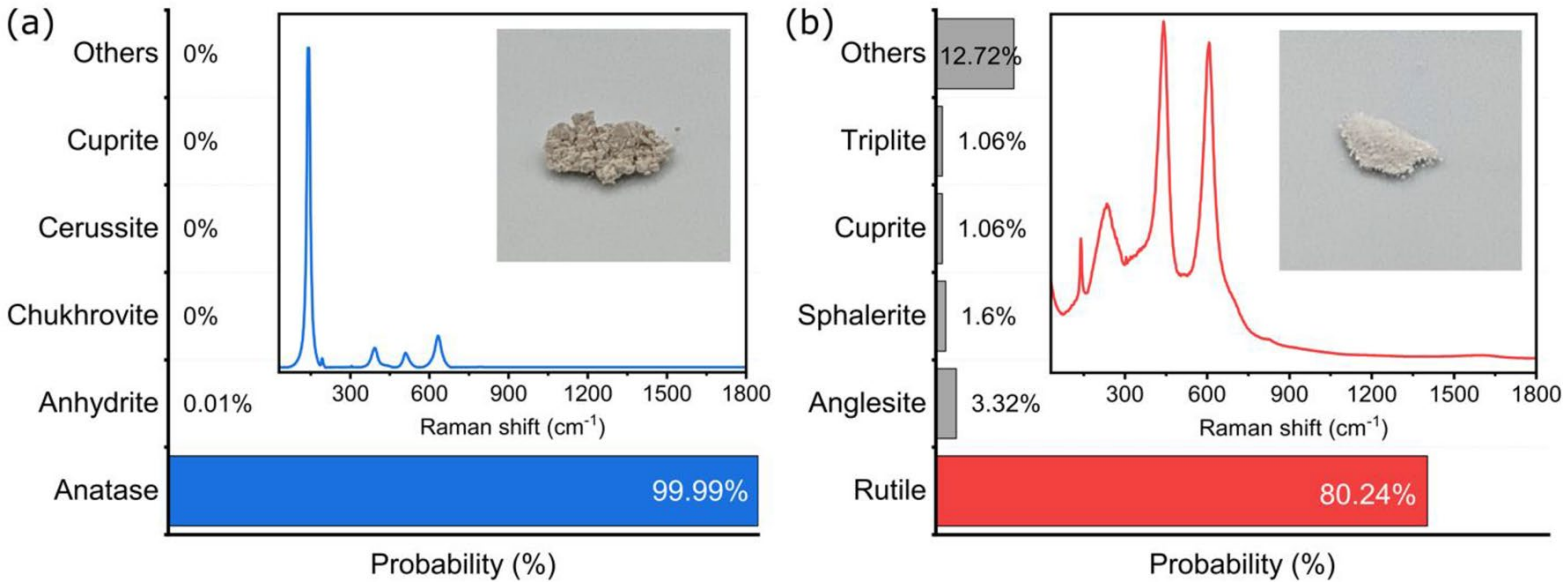
(**a**) The figure shows the response of the model to our synthesized anatase TiO_2_ sample. The model can identify the presence of anatase with utmost certainty. (**b**) The figure shows the response of the model to our rutile TiO_2_ sample. The model correctly identifies Rutile with a high degree of confidence. The Raman spectrum of the mineral and the image of the sample are presented to provide adequate context. The bar chart shows the Top-5 predictions and the other predictions are combined as others.

The Fig. 4a,b shows the predictions from the model. Our model correctly identifies both anatase (Fig. 4a) and rutile (Fig. 4b) from our pure samples with a high degree of confidence. It yields a 80.24% percent confidence for the rutile TiO_2_ sample, compared with 99.99% for the anatase TiO_2_ sample.

**Figure 5:**
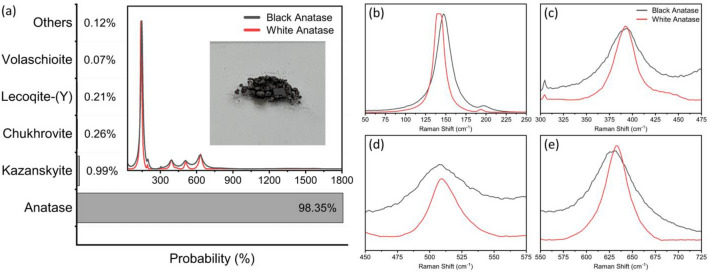
(**a**) The figure shows the response of the model to defect-rich black Anatase. (**b**–**e**) shows a detailed comparative view of the four main peaks in the Raman spectra of defect rich black Anatase and Standard white Anatase. The model correctly identifies Anatase in the modified spectra with a slightly lower level of confidence. The ability of the model to correctly characterise the modified spectrum shows the generalisation power of the model. The Raman spectrum of the mineral and the image of the sample are presented to provide adequate context. The bar chart shows the Top-5 predictions and the other predictions are combined as others.

The presence of surface defects on the crystalline lattice of black anatase shifts the Raman spectrum^56^. This presents an interesting challenge for the model. We find that the model is able to identify Anatase in the modified Spectrum. Moreover, it assigns a lower probability to the presence of Anatase which may indicate the presence of defects in the crystalline structure. It is pertinent to point out that even though the model was not exposed to the modified spectrum of black anatase during training, the model is able to recognise it as Anatase. This demonstrates an ability of the model to generalise. The Fig. 5a–e shows the prediction of the model given the spectrum of black anatase and presents a comparative view of the Raman spectra.

**Figure 6:**
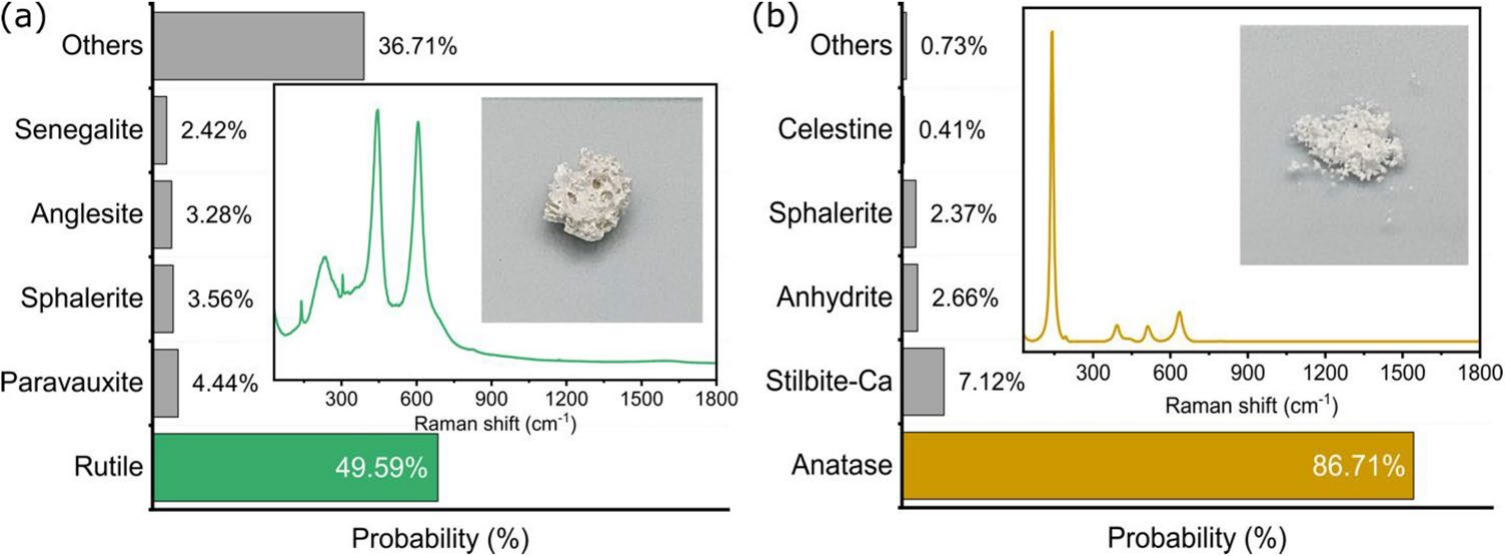
(**a**) The figure shows the response of the model to TiO_2_ at 800 °C, below the 1100 °C temperature allowing full-conversion to rutile. The model still identifies rutile with a confidence of 49.59% suggesting an intermediate state with incomplete conversion. (**b**) The figure shows the response of the model to Degussa, P25. Degussa contains more than 70% anatase and around 20% rutile and amorphous compounds. The model can identify anatase with a lower confidence (86.71%). The Raman spectrum of the mineral and the image of the sample are presented to provide adequate context. The bar chart shows the top 5 predictions and the other predictions are combined as others.

We also wanted to evaluate our model’s ability to recognize the differences in crystalline structure for TiO_2_ in an intermediate phase between anatase and rutile. To do so, a sample was prepared by annealing at 800 °C. The TiO_2_ fully converts from anatase to rutile at 1100 °C. At 800 °C, the crystalline structure resembles rutile, but the conversion is not complete^69^. The Fig. 6a shows the measurements for rutile TiO_2_ annealed below the full-conversion temperature. Here, the model still predicts rutile but with a lower confidence level since the Raman spectrum is significantly different from the fully-converted rutile. As such, we believe that the probability value could potentially be also be used as an indicator for impurities and defects in the crystal structure. However, this would require more extensive studies well beyond the scope of this work.

Finally, commercial Degussa (Evonik) P25, Aeroxide TiO_2_ is a titania photocatalyst that is widely used because of its relatively high levels of activity in many photocatalytic reactions systems^70^. The literature shows that this P25 contains more than 70% anatase TiO_2_, with significantly lower amounts of rutile and amorphous TiO_2_ powders^61^. Figure 6b indicates a dominant anatase Raman structure, with some contributions of the rutile TiO_2_ with peaks around 445 cm^−1^ and 611 cm^−1^. Accordingly, our model successfully detects the dominant anatase TiO_2_ in the commercial P25 powder. However, the small features at 200 cm^−1^ and 450 cm^−1^ are consistent with the presence of much smaller concentrations of rutile TiO_2_ powder. As a result, the model detects anatase with a lower confidence (86.71%). However, our model is unable to detect the presence of rutile in the spectrum. Once again, we believe that future studies could exploit this lower probability and use it as a good indicator to detect the presence of contaminants or defects in the pure compound.

**Figure 7:**
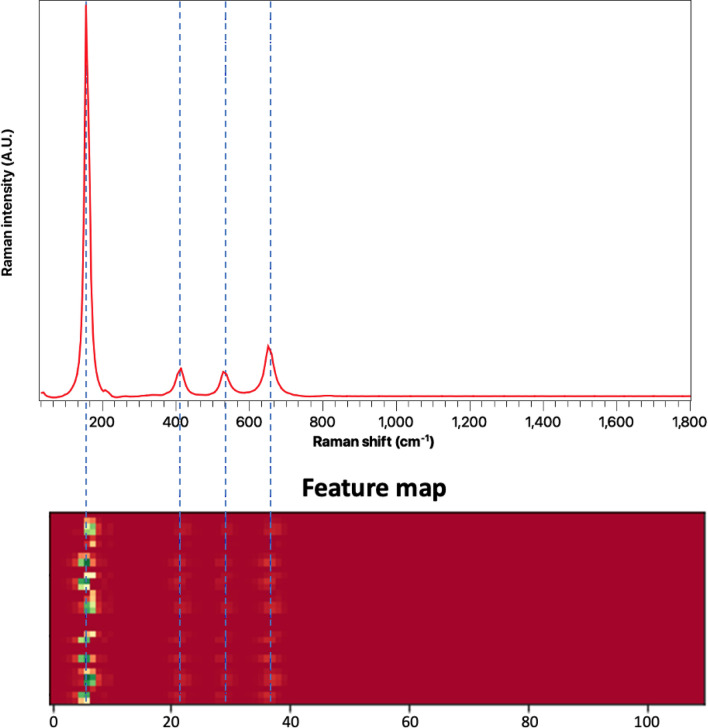
The figures shows the feature maps learned by the model while analysing the Raman spectrum of white anatase powder annealed at 500 °C. The initial layers recognise a faint representation of the high intensity peak at 150 cm^−1^. However, deeper layers help clearly recognize the other peaks.

Neophytes sometime describe neural networks as *black-box* models. However, we believe that it is important to look under the hood to achieve a deeper understanding and visualize the patterns recognized by the neural network. Feature maps have been previously used to visualize the representations learned by the neural network^71^. We plot the feature maps learned by our model at each layer to show the progression of features learned by the model while analysing the spectrum of our pure white anatase powder sample. Figure 7 shows that initially the model faintly recognizes the high intensity peak at 150 cm^−1^. However, as we go deeper, the model learns to recognize the other peaks. This enables the model to correctly identify the compound with a higher confidence. The feature maps for all the layers of the proposed model are provided in the supplementary information.

To further investigate the effect of noise and integration time on the model's performance, we conduct a sensitivity analysis as described in the following section.

### Sensitivity analysis

Performing high-quality Raman micro-spectroscopy analysis on our WITec instrument requires a relatively-high level of expertise to optimize the different instrument and software parameters. This high-end equipment allows the expert to adjust the spectrum by controlling a wide range of parameters. For our sensitivity analysis, we fixed the accumulations at 100 scans and varied the integration time and intensity. The *integration time* defines how long the spectrometer's detector collects light. The longer the integration time, the stronger the signal. However, an excessively long integration time may cause the detector to saturate, which can result in clipping of the peak and spectrum distortion. The same goes for the stray light entering the spectrometer.

We acquired the Raman spectrum for both the pure anatase and rutile samples at three integration times, namely 1 s, 5 s and 10 s. As expected, we observe that the signal-to-noise ratio (SNR) in the spectrum decreases as we reduce the integration time. Even for the 10 s integration time, we did not observe any saturation at the detector.

**Figure 8:**
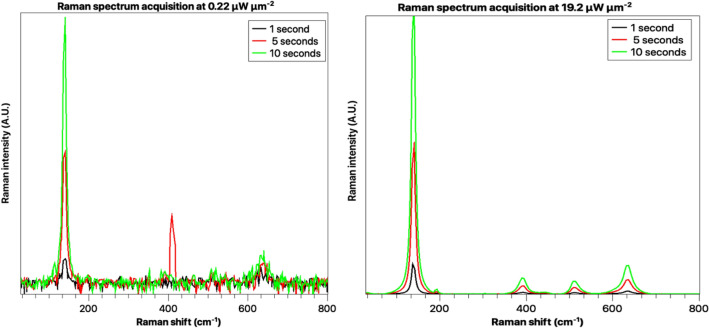
The figure shows the anatase spectrum at different excitation power densities and different integration times. Increasing integration time and the incident excitation power improves the SNR, which improves the spectrum’s quality.

The intensity of the laser can also be varied by varying the built-in variable attenuator. For the analysis, we experimented with different intensities and found that fixing the attenuator to a lower excitation power setting results in a noisy spectrum. If we gradually increase the excitation power, we can observe a much cleaner spectrum with a higher signal-to-noise (SNR). The Fig. 8 shows the effect of varying the integration time and excitation power density on the Raman spectrum of our pure white anatase sample. The detailed results of the sensitivity analysis are provided in the supplementary information.

There, we observe that the model is far less robust to high noise levels. We observe that reducing the SNR by significantly reducing the excitation power density or the integration time directly leads to lower confidence or even incorrect predictions. We believe that excessive noise reduces the uniqueness of the spectrum, thus increasing the level of confusion for the model. We have presented a study of the misclassification of the noisy Raman Spectrum of the Anatase polymorph in the supplementary information. Several researchers have studied the use of machine learning models for improving the signal-to-noise ratio of the Raman spectrum^72,73^. We believe that the use of these models in conjunction with our proposed model will address the above-mentioned challenge.

In contrast, we also observe that increasing the SNR significantly helps the model correctly identify the sample. Thus, users of our model are recommended to ensure that the acquisition of the sample is done at optimal excitation power densities and integration times. Most experienced material scientists, chemists and chemical engineers regularly use complementary material characterization tools such as FTIR or XRD to correctly identify compounds. In the near future, we believe that the proposed deep-learning approach could potentially be augmented by adding information from FTIR or XRD for identifying the compounds with even better accuracy using fully-automated multi-modal analysis.

## Conclusion

This paper presents a deep-learning framework to accurately identify the mineral compounds from their Raman spectra. Our proposed framework can accurately identify both raw and pre-processed Raman data. The model is lightweight and can achieve a Top-1 accuracy reaching 99.12% on the test samples. Experimental evaluation was also performed using TiO_2_ powders. For the experimental validation in the laboratory, we synthesized white anatase and rutile using standard procedures from the literature. The model was able to accurately identify both polymorphs from the Raman spectrum.

Furthermore, we evaluated the model for more complex TiO_2_ samples such as intermediate phases obtained with a lower annealing temperature (so-called mixed-phase TiO_2_). The TiO_2_ powder in an intermediate (mixed) phase was correctly identified as rutile with a lower confidence (probability). With more extensive work, we believe that the lower probability can be potentially used as a quantitative indicator to evaluate the presence of surface defects or impurities in more complex samples.

We also carried out extensive ablation studies by modifying/removing components of the model. We observe that the proposed architecture with the LSTM module and ReLU activation function clearly provides the best performances. We also evaluated the performances of the model using noisy data, obtained by varying the integration time and excitation power density during acquisition of the Raman spectra. We find that our model performs best with low-noise samples. Therefore, optimizing the excitation power and the integration time significantly helps the model correctly identify the compounds from the Raman data. While we believe it provides a major breakthrough in the analysis of complex materials and compounds, we believe that leveraging information from other characterization techniques (multimodal analysis) could further increase the model’s performance and remove most misclassification issues.

## Supplementary Information

Supplementary Information.

## Data Availability

The datasets generated and/or analyzed during the current study are available in the RRUFF data repository (https://rruff.info/).

## References

[1] Larkin P (2017). Infrared and Raman Spectroscopy: Principles and Spectral Interpretation.

[2] Meza Ramirez CA, Greenop M, Ashton L, Rehman IU (2021). Applications of machine learning in spectroscopy. Appl. Spectrosc. Rev..

[3] Penido CA, Pacheco MTT, Zângaro RA, Silveira L (2015). Identification of different forms of cocaine and substances used in adulteration using near-infrared raman spectroscopy and infrared absorption spectroscopy. J. Forensic Sci..

[4] Castro K, Pérez-Alonso M, Rodríguez-Laso M, Fernández LÁ, Madariaga J (2005). On-line FT-Raman and dispersive Raman spectra database of artists’ materials (e-VISART database). Anal. Bioanal. Chem..

[5] Zhang Z-M (2010). An intelligent background-correction algorithm for highly fluorescent samples in Raman spectroscopy. J. Raman Spectrosc..

[6] Gonzalez Zelaya, C. V. Towards explaining the effects of data preprocessing on machine learning. In *2019 IEEE 35th International Conference on Data Engineering (ICDE)* 2086–2090 10.1109/ICDE.2019.00245 (2019).

[7] Bocklitz T, Walter A, Hartmann K, Rösch P, Popp J (2011). How to pre-process Raman spectra for reliable and stable models?. Anal. Chim. Acta.

[8] Jehlička J, Culka A, Bersani D, Vandenabeele P (2017). Comparison of seven portable Raman spectrometers: Beryl as a case study. J. Raman Spectrosc..

[9] Chandler, L., Huang, B. & Mu, T. T. A smart handheld Raman spectrometer with cloud and AI deep learning algorithm for mixture analysis. In *Next-Generation Spectroscopic Technologies XII*, vol. 10983, 1098308 (International Society for Optics and Photonics, 2019).

[10] Jermyn M (2017). Highly accurate detection of cancer in situ with intraoperative, label-free, multimodal optical spectroscopy. Can. Res..

[11] McDevitt N, Donley M, Zabinski J (1993). Utilization of Raman spectroscopy in tribochemistry studies. Wear.

[12] Khan H, Berk D (2014). Effect of a chelating agent on the physicochemical properties of TiO_2_: Characterization and photocatalytic activity. Catal. Lett..

[13] Benavides JA, Trudeau CP, Gerlein LF, Cloutier SG (2018). Laser selective photoactivation of amorphous TiO_2_ films to anatase and/or rutile crystalline phases. ACS Appl. Energy Mater..

[14] Dong F (2015). Surface oxygen-vacancy induced photocatalytic activity of La(OH)_3_ nanorods prepared by a fast and scalable method. Phys. Chem. Chem. Phys..

[15] Wang D, Bierwagen GP (2009). Sol–gel coatings on metals for corrosion protection. Prog. Org. Coat..

[16] Gong M, Li Y, Guo Y, Lv X, Dou X (2018). 2D TiO_2_ nanosheets for ultrasensitive humidity sensing application benefited by abundant surface oxygen vacancy defects. Sens. Actuators B Chem..

[17] Stathopoulos S (2017). Multibit memory operation of metal-oxide bi-layer memristors. Sci. Rep..

[18] Pacchioni G (2003). Oxygen vacancy: The invisible agent on oxide surfaces. ChemPhysChem.

[19] Bengio Y, LeCun Y (2007). Scaling learning algorithms towards AI. Large-scale Kernel Mach..

[20] Grotch SL (1970). Matching of mass spectra when peak height is encoded to one bit. Anal. Chem..

[21] Knock B, Smith I, Wright D, Ridley R, Kelly W (1970). Compound identification by computer matching of low resolution mass spectra. Anal. Chem..

[22] Kotsiantis SB, Zaharakis I, Pintelas P (2007). Supervised machine learning: A review of classification techniques. Emerg. Artif. Intell. Appl. Comput. Eng..

[23] Khanum M, Mahboob T, Imtiaz W, Ghafoor HA, Sehar R (2015). A survey on unsupervised machine learning algorithms for automation, classification and maintenance. Int. J. Comput. Appl..

[24] Patra BK, Nandi S, Viswanath P (2011). A distance based clustering method for arbitrary shaped clusters in large datasets. Pattern Recogn..

[25] Ghodsi A (2006). Dimensionality reduction a short tutorial. Dept. Stat. Actuarial Sci. Univ. Waterloo Ontario Canada.

[26] Sevetlidis V, Pavlidis G (2019). Effective Raman spectra identification with tree-based methods. J. Cult. Herit..

[27] Yang L, Jin R (2006). Distance metric learning: A comprehensive survey. Michigan State Univ..

[28] Roselli, D., Matthews, J. & Talagala, N. Managing bias in AI. In *Companion Proceedings of The 2019 World Wide Web Conference* 539–544 (Association for Computing Machinery, 2019). 10.1145/3308560.3317590.

[29] Whang SE, Lee J-G (2020). Data collection and quality challenges for deep learning. Proc. VLDB Endow..

[30] Hastie, T., Tibshirani, R. & Friedman, J. Overview of supervised learning. In *The Elements of Statistical Learning* 9–41 (Springer, 2009).

[31] Carey C, Boucher T, Mahadevan S, Bartholomew P, Dyar MD (2015). Machine learning tools formineral recognition and classification from Raman spectroscopy. J. Raman Spectrosc..

[32] Mao Y (2020). Machine learning analysis of Raman spectra of MoS2. Nanomaterials.

[33] Hearst MA, Dumais ST, Osuna E, Platt J, Scholkopf B (1998). Support vector machines. IEEE Intell. Syst. Appl..

[34] Huang X, Maier A, Hornegger J, Suykens JA (2017). Indefinite kernels in least squares support vector machines and principal component analysis. Appl. Comput. Harmon. Anal..

[35] Liu J (2017). Deep convolutional neural networks for Raman spectrum recognition: A unified solution. Analyst.

[36] Liu J, Gibson SJ, Mills J, Osadchy M (2019). Dynamic spectrum matching with one-shot learning. Chemom. Intell. Lab. Syst..

[37] Salman, S. & Liu, X. Overfitting mechanism and avoidance in deep neural networks. *arXiv preprint* arXiv:1901.06566 (2019).

[38] Chen X (2019). Fast and accurate decoding of Raman spectra-encoded suspension arrays using deep learning. Analyst.

[39] Malek S, Melgani F, Bazi Y (2018). One-dimensional convolutional neural networks for spectroscopic signal regression. J. Chemom..

[40] Lafuente, B., Downs, R. T., Yang, H. & Stone, N. 1. The power of databases: The RRUFF project. In *Highlights in Mineralogical Crystallography* 1–30 (De Gruyter (O), 2015).

[41] Fan X, Ming W, Zeng H, Zhang Z, Lu H (2019). Deep learning-based component identification for the Raman spectra of mixtures. Analyst.

[42] Zhao, W. Research on the deep learning of the small sample data based on transfer learning. In *AIP Conference Proceedings* vol. 1864, 020018 (AIP Publishing LLC, 2017).

[43] Bjerrum, E. J., Glahder, M. & Skov, T. Data augmentation of spectral data for convolutional neural network (CNN) based deep chemometrics. *arXiv preprint* arXiv:1710.01927 (2017).

[44] Zhang R (2020). Transfer-learning-based Raman spectra identification. J. Raman Spectrosc..

[45] Lieber CA, Mahadevan-Jansen A (2003). Automated method for subtraction of fluorescence from biological Raman spectra. Appl. Spectrosc..

[46] Liu, Y. & Yu, Y. A survey of the baseline correction algorithms for real-time spectroscopy processing. In *Real-time Photonic Measurements, Data Management, and Processing II* vol. 10026, 100260Q (International Society for Optics and Photonics, 2016).

[47] Hochreiter S, Schmidhuber J (1997). Long short-term memory. Neural Comput..

[48] Baldi, P. & Sadowski, P. J. Understanding dropout. in *Advances in Neural Information Processing Systems* 26, (2013).

[49] Ruder, S. An overview of gradient descent optimization algorithms. *arXiv preprint* arXiv:1609.04747 (2016).

[50] Abadi, M. *et al.* TensorFlow: a system for large-scale machine learning. In *12th USENIX Symposium on Operating Systems Design and Implementation (OSDI 16)* 265–283 (2016).

[51] Tharwat A (2020). Classification assessment methods. Appl. Comput. Inform..

[52] Keskar, N. S. & Socher, R. Improving generalization performance by switching from adam to sgd. *arXiv preprint* arXiv:1712.07628 (2017).

[53] Robel I, Subramanian V, Kuno M, Kamat PV (2006). Quantum dot solar cells. Harvesting light energy with CdSe nanocrystals molecularly linked to mesoscopic TiO_2_ films. J. Am. Chem. Soc..

[54] O’Regan B, Grätzel M (1991). A low-cost, high-efficiency solar cell based on dye-sensitized colloidal TiO_2_ films. Nature.

[55] Gerlein LF, Benavides-Guerrero JA, Cloutier SG (2019). Laser-assisted, large-area selective crystallization and patterning of titanium dioxide polymorphs. Adv. Eng. Mater..

[56] Benavides-Guerrero JA (2022). Synthesis of vacancy-rich titania particles suitable for the additive manufacturing of ceramics. Sci. Rep..

[57] Vorkapic D, Matsoukas T (1998). Effect of temperature and alcohols in the preparation of titania nanoparticles from alkoxides. J. Am. Ceram. Soc..

[58] Iwana BK, Uchida S (2021). An empirical survey of data augmentation for time series classification with neural networks. PLoS ONE.

[59] Schafer RW (2011). What is a Savitzky-Golay filter?[lecture notes]. IEEE Signal Process. Mag..

[60] Baek S-J, Park A, Ahn Y-J, Choo J (2015). Baseline correction using asymmetrically reweighted penalized least squares smoothing. Analyst.

[61] Ohtani B, Prieto-Mahaney OO, Li D, Abe R (2010). What is Degussa (Evonik) P25? Crystalline composition analysis, reconstruction from isolated pure particles and photocatalytic activity test. J. Photochem. Photobiol., A.

[62] Ibtehaz, N. *et al.* RamanNet: a generalized neural network architecture for Raman spectrum analysis. *arXiv preprint* arXiv:2201.09737 (2022).

[63] Sang X, Zhou R, Li Y, Xiong S (2022). One-dimensional deep convolutional neural network for mineral classification from Raman spectroscopy. Neural Process. Lett..

[64] Zhou W, Tang Y, Qian Z, Wang J, Guo H (2022). Deeply-recursive convolutional neural network for Raman spectra identification. RSC Adv..

[65] Afendras G, Markatou M (2019). Optimality of training/test size and resampling effectiveness in cross-validation. J. Stat. Plan. Inference.

[66] Yu Y, Si X, Hu C, Zhang J (2019). A review of recurrent neural networks: LSTM cells and network architectures. Neural Comput..

[67] Bengio, Y. *Learning Deep Architectures for AI*. 56.

[68] Rasamoelina, A. D., Adjailia, F. & Sinčák, P. A review of activation function for artificial neural network. In *2020 IEEE 18th World Symposium on Applied Machine Intelligence and Informatics (SAMI)* 281–286 (IEEE, 2020).

[69] Ricci PC (2013). Anatase-to-rutile phase transition in TiO_2_ nanoparticles irradiated by visible light. J. Phys. Chem. C.

[70] Janus M, Morawski A (2007). New method of improving photocatalytic activity of commercial Degussa P25 for azo dyes decomposition. Appl. Catal. B.

[71] Qin, Z., Yu, F., Liu, C. & Chen, X. How convolutional neural network see the world—A survey of convolutional neural network visualization methods. *arXiv preprint* arXiv:1804.11191 (2018).

[72] Zhao XY, Liu GY, Sui YT, Xu M, Tong L (2021). Denoising method for Raman spectra with low signal-to-noise ratio based on feature extraction. Spectrochim. Acta Part A Mol. Biomol. Spectrosc..

[73] Pan, L. *et al.* Noise Reduction Technique for Raman Spectrum using Deep Learning Network. Preprint at arXiv:2009.04067 (2020).

