# Supplementary material for: Deep-learning framework for fully-automated recognition of TiO2 polymorphs based on Raman spectroscopy
Source: Sci Rep. 2022 Dec 19;12:21874. doi: 10.1038/s41598-022-26343-3 (PMC9763332; doi:10.1038/s41598-022-26343-3)
Supplement: Supplementary file 1 — Supplementary Information. [file 41598_2022_26343_MOESM1_ESM.docx]

**Supplementary:** **Deep-learning framework for fully-automated recognition of TiO_2_ polymorphs based on Raman Spectroscopy**

Abhiroop Bhattacharya, Jaime A. Benavides, Luis Felipe Gerlein, Sylvain G. Cloutier*

Department of Electrical Engineering, École de technologie supérieure,

1100 Notre-Dame West, Montreal (QC), Canada H3C 1K3

E-mail: [sylvaing.cloutier@etsmtl.ca](mailto:sylvaing.cloutier@etsmtl.ca)

**Model Predictions**

Our model is able to correctly identify both Anatase and Rutile TiO_2_ from a pure sample of the compound. The model associates a probability of 80% percent for the rutile sample. For Anatase, the model is able to predict the correct compound with 99% percent probability. The Figure S1 and Figure S2 show the predictions from the model.

**Model's response to pure Anatase TiO_2_ sample**


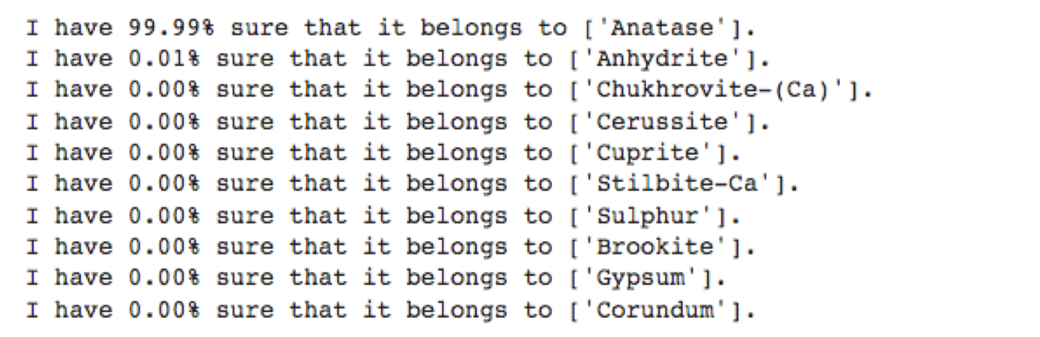


**Figure S1. The figure shows the response of the model to the pure Anatase sample. The model can clearly identify the pure Anatase with certainity . This is due to the clear dominant peak in the Raman spectrum at 150 cm^-1^ and the high SNR of the sample.**

**Model's response to pure Rutile TiO_2_ sample**

**Figure S2. The figure shows the response of the model to the pure Rutile sample. The model can identify the Rutile with a high level of confidence (probability).**

The Figure S3 shows the model’s response to the Raman signature of the defect rich black Anatase sample. The model is able to correctly detect Anatase based on the modified spectrum.

**Model's response to defect rich black Anatase sample**


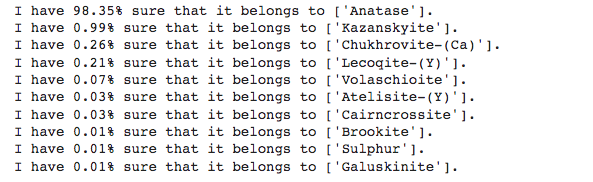


**Figure S3. The figure shows the response of the model to the defect rich Black Anatase Ramans signature. The model can identify Anatase with a slightly lower confidence.**

The Figure S4 shows the measurements for the Ti0_2_ sample in the intermediate (mixed) phase at after annealing at 800 ^o^C.

**Model's response to intermediate (mixed-phase) TiO_2_ sample**

**Figure S4. The figure shows the response of the model to Ti0_2_ in the intermediate (mixed) phase after annealing at 800 ^o^C. The model can identify rutile with a probability of 49.59%, suggesting significant changes in the crystalline structure.**

Degussa (Evonik) P25, Aeroxide TiO_2_ P 25, is a titania photocatalyst that is used widely because of its relatively high levels of activity in many photocatalytic reactions systems. The Figure S5 shows the response of our model to DEGUSSA P25 sample.

**Model's response to commercial DEGUSSA P25 TiO_2_ sample**


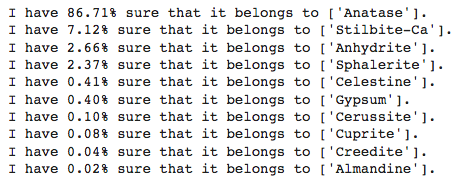


**Figure S5. The figure shows the response of the model to commercial Degussa, P25. Degussa contains more than 70% Anatase and around 20% rutile and amorphous compounds. The model can identify Anatase with a probability of 86.71%, indicating the dominant presence of Anatase but with a contamination by other compounds.**

**Misclassification**

In some rare instances our model associated a high probability (suggesting a high level of confidence) to an incorrect prediction. For example, Berborite is a beryllium borate mineral with the chemical formula Be_2(_BO_3_) (OH, F) (H_2_O). It is colorless and leaves a white streak. As shown in the figure S6, the Raman spectrum of Berborite has dominant peaks around 1000 cm^-1^, 800 cm^-1^ 380 cm^-1^ and 400 cm^-1^. However, the model misclassifies this Berborite spectra as Chukhrovite-(Ca). This is a halide mineral with the chemical formula Ca₃Ca₁₅Al₂SO₄F₁₃12H₂O. The Raman spectrum shows that it has a strong dominant peak at the exact same position near 1000 cm^-1^ and smaller peaks from 200 cm^-1^ to 600 cm^-1^. The model compares the samples at high intensity and it is confused by the smaller peaks. However, the same strong dominant peak falsely provides a greater confidence to the model in its prediction. The Raman spectra of Berborite and Chukhrovite are compared below.

**
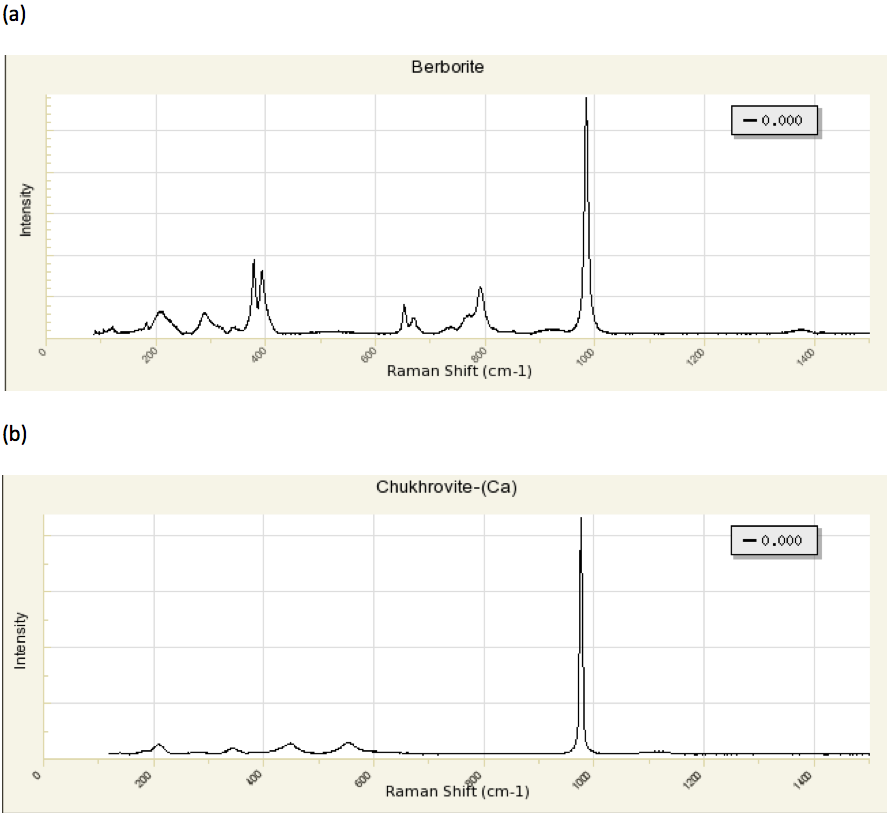
**

**Figure S6. The figure present a comparative picture of the Raman spectrum of (a)Berborite and (b) Chukhrovit(Ca). Our model misclassifies Berborite as Chukhrovite (Ca) due to the similarity in the Raman spectrum. The strong dominant peak just below 1000 cm-1 is present**

**Sensitivity Analysis**

We observed that varying the intensity and integration time affects the Signal to Noise (SNR) ratio of the acquired Raman spectrum. For the Anatase polymorph, we observed that the probability of the model correctly identifying the mineral decreases with decrease in the integration time and intensity of the laser. For a low SNR, the Raman spectrum of the noisy Anatase sample has only one clear dominant peak. As a result, the model may misclassify the noisy spectrum as Sinoite (Si_2_N_2_O) due to the presence of a dominant high-intensity peak at 150 cm^-1^ for both materials (Figure S7).


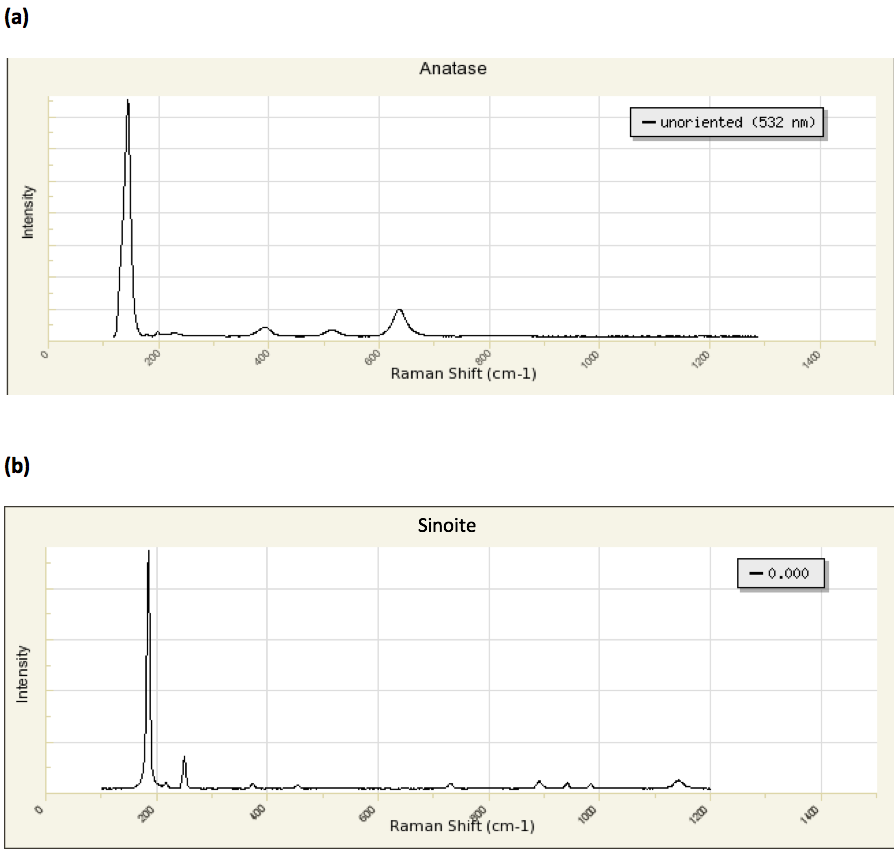
**Figure S7. The figure presents a comparative picture of the Raman spectrum of (a)Anatase and (b) Sinoite. Our model misclassifies a noisy Anatase spectrum as Sinoite due to the similarity in the Raman spectrum. The strong dominant peak around 150 cm-1**

**Model Hyperparameters**

The hyperparameters (Table S1) used in the model are given below

| **Hyperparameters** | **Value** |
| --- | --- |
| Epochs | 100 |
| Initial learning rate | 1e-3 |
| Learning Rate Schedule Factor | 0.1 |
| Learning Rate Schedule Patience | 2 |
| Min Learning Rate | 1e-5 |
| Early stopping Patience | 5 |
| Convolutional Drop out | 0.2 |
| Dense Drop Out | 0.5 |

**Table S1. The table outlines the hyperparameters used in the model.**

**Model Diagram**

The block diagram in figure S8 presents a detailed diagram of the proposed model. The block diagram clearly shows the number of filters and the shape of the input and output tensors of each layer of the model. The model has four 1D Convolutional layers with a kernel size of 2 and *same* padding. The max pooling layers have a pool size of 2 and a stride of 2.


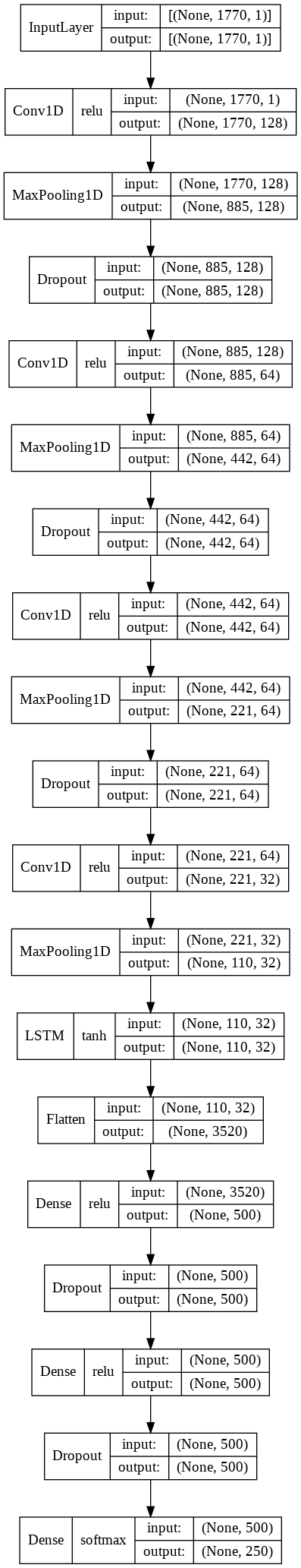


**Figure S8. The block diagram shows a detailed description of the proposed model.**

The feature map of each layer of the network has been extracted from the trained model. The feature maps have been plotted using the 'RdYlGn' colour map in the figure S9. We can clearly observe that as the model gets deeper it is able to encode the distinguishing features of the spectra.


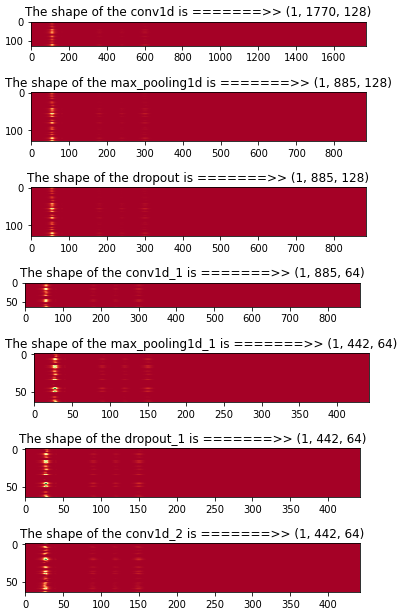


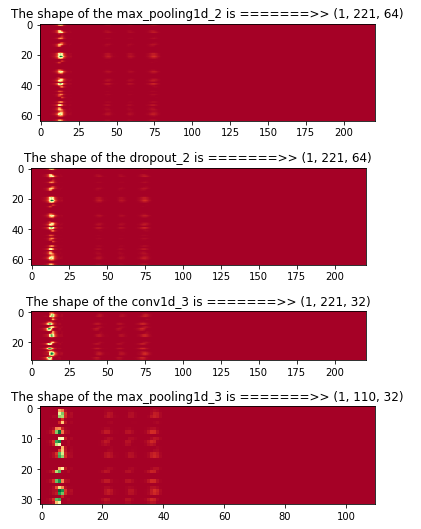


**Figure S9. The feature maps extracted for each layer of the deep learning model.**

**Analysis of the RRUFF Dataset**

The table S2 presents the analysis of the of the Raman spectra of the RRUFF Dataset.

| **Sample Quality** | **Oriented** | | **Unoriented** | |
| --- | --- | --- | --- | --- |
|  | **Processed** | **Raw** | **Processed** | **Raw** |
| Excellent | 3696 | 0 | 5256 | 11 |
| Fair | 9 | 1 | 1394 | 6 |
| Poor | 0 | 1 | 0 | 0 |
| Unrated | 1977 | 5757 | 370 | 7855 |

**Table S2. The table captures the distribution of samples in the RRUFF dataset.**
